# Developing of Focal Ischemia in the Hippocampus or the Amygdala Reveals a Regional Compensation Rule for Fear Memory Acquisition

**DOI:** 10.1523/ENEURO.0398-20.2021

**Published:** 2021-04-21

**Authors:** Cheng-Long Yu, Jin-Nan Li, Ping Gan, Li-Ping Wang, Yue-Xiong Yang, Da-Fu Yu, Rong-Rong Mao, Fu-Qiang Xu, Qi-Xin Zhou, Gal Richter-Levin, Lin Xu, Heng Zhou

**Affiliations:** 1 Kunming Institute of Zoology and Key Laboratory of Animal Models and Human Disease Mechanisms, Chinese Academy of Sciences (CAS), Kunming 650223, China; 2 Department of Biochemistry and Molecular Biology, College of Basic Medicine, Kunming Medical University, Kunming, 650500, China; 3 Department of Nuclear Medicine, First People’s Hospital of Yunnan Province, and Key Laboratory of Medical Imaging, Kunming University of Science and Technology, Kunming 650032, China; 4 Wuhan Institute of Physics and Mathematics, CAS, Wuhan 430071, China; 5 Department of Neurobiology and Ethology, and Department of Psychology, University of Haifa, Haifa 3498838, Israel; 6 The Brain Cognition and Brain Disease Institute (BCBDI), Shenzhen Institutes of Advanced Technology, Chinese Academy of Sciences, Shenzhen, 518055, China

**Keywords:** amygdala, circuits, fear memory, hippocampus, ischemia

## Abstract

Circuit compensation is often observed in patients with acute ischemic stroke, suggesting the importance of the interaction between brain regions. Also, contextual fear memory is an association between multisensory contexts and fearful stimuli, for which the interaction between the hippocampus and the amygdala is believed to be critical. To understand how focal ischemia in one region could influence the other region, we used a modified photo-thrombosis to induce focal ischemia in the hippocampus or the amygdala or both in freely-moving rats. We found that the learning curve and short-term memory (STM) were not affected in the rats although focal ischemia was induced 5 h before learning in either the hippocampus or the amygdala; these were impaired by the induction of ischemia in both the regions. Furthermore, the learning curve and STM were impaired when ischemia was induced 24 h before learning in either the hippocampus or the amygdala when the synaptic transmission was altered in one region because of ischemia in the other region. These results suggest that the circuit compensation between the hippocampus and the amygdala is critical for fear memory acquisition.

## Significance Statement

Contextual fear memory needs the interconnection between the hippocampus and the amygdala. However, it is unclear whether and how the two regions produce circuit compensation under an ischemic situation. Here, we employed the developing of ischemia in the hippocampus or the amygdala or both in freely moving rats. We found that memory acquisition was not affected 5 h postischemia, but it was impaired 24 h postischemia when the synaptic transmission was impaired in one region because of ischemia in the other region. Furthermore, ischemia in both brain regions impaired memory acquisition. These results indicate a circuit compensation between the hippocampus and the amygdala in memory acquisition if one with ischemia does not affect the function of the other.

## Introduction

Fear conditioning is a type of Pavlovian learning that builds a rapid association between neutral and fearful stimuli. It depends on the neural network that engages cortical and subcortical regions such as the hippocampus and the amygdala (Rogan and LeDoux, 1996). Previous kinds of literature have established that the hippocampus is important for contextual fear memory while the amygdala is critical for cued fear memory (Rogan and LeDoux, 1996; Gräff et al., 2014). However, other reports suggest that contextual or cued fear memory is dependent on the interconnection between the hippocampus and the amygdala, such as their dynamic interaction and/or enhanced coherence (Selden et al., 1991; Richter-Levin and Akirav, 2000; Seidenbecher et al., 2003; Maren et al., 2013).

The interconnection of the hippocampus and the amygdala would involve the context information processed in the hippocampus and that is associated with negative information processed by the basolateral amygdala (BLA) during fear conditioning, and the activated BLA will trigger the freezing behavior through the relay circuit containing the central nucleus of the amygdala and the ventral periaqueductal gray (Miserendino et al., 1990; Fendt and Fanselow, 1999; Wilensky et al., 2006). Either pharmacological inactivation or lesion of the hippocampus leads to the impaired contextual fear memory (Remaud et al., 2014; Zhou et al., 2016). While studies with excitotoxic manner indicate that hippocampal lesion has no effect on the contextual fear formation (Maren et al., 1997; Cho et al., 1999; Richmond et al., 1999; Rudy et al., 2002; Zhou et al., 2016). The lesion or inactivation of the BLA impairs the contextual fear acquisition (Phillips and LeDoux, 1992; Helmstetter and Bellgowan, 1994), which can be overcome by extensive training (Maren, 1999; Gale et al., 2004; Wiltgen et al., 2006; Ponnusamy et al., 2007). These pieces of evidence suggest that under some circumstances, contextual or cued fear memory can be acquired through the alternate neural pathways, which could be explained by circuitry compensation when the regular one is impaired (Maren et al., 1997; Poulos et al., 2010; Zelikowsky et al., 2012). Interestingly, the circuitry compensation only happens in the animal under the damage over 7 d anterograde lesions but not with the acute pharmacological blockade (Zelikowsky et al., 2012; Zhou et al., 2016, 2017). This suggests that the damage to the neural pathway in a developing or not excessive manner should be the prerequisites for circuit compensation, as it can provide the essential spatial or temporal frame for the remodeling for information process in alternate pathways.

Circuit compensation is also frequently observed in patients with acute ischemic stroke, for which the function of one brain region with ischemia is impaired initially but it is recovered later because of compensation of other brain regions without ischemia. Thus, we suspected that circuit compensation may be different from that using pharmacological lesion or inhibition of a brain region. In our previous study, we modified a photo-thrombosis model of acute ischemic stroke that enabled us to induce focal ischemia in freely moving rodents. This model allows us to explore circuit compensation between the hippocampus and the amygdala for contextual fear memory acquisition under an ischemic situation.

## Materials and Methods

### Animals

Adult male Sprague Dawley rats weighing 250–350 g and three-month C57/BL6 mice were used in this study. Animals were group-housed while single after surgery, in ventilated cages with free access to food and water in a temperature-regulated environment with a 12/12 h light/dark cycle in the animal housing center. All experimental protocols were approved by the animal ethics committee.

### Surgery and cannula implantation

Surgery was performed on rats under pentobarbital anesthesia (60 mg/kg, i.p.; Sigma). Animals were ventilated with 95% O_2_/5% CO_2_ through a mask and positioned in a stereotaxic frame. Two stainless-steel guide cannulas (26 G) were bilaterally implanted 1 mm above the dorsal hippocampus or/and amygdala based on the Paxinos and Watson rat brain atlas: the coordinates of the hippocampus were anteroposterior (AP) −3.8 mm, mediolateral (ML) ±2.8 mm, and dorsoventral (DV) −3.0 mm; those of the amygdala were AP −2.8 mm, ML ±4.8 mm, and DV −8.0 mm, from the bregma. A guide cannula was affixed to the skull by using dental cement. A stylet was introduced into the guide cannula to prevent obstruction.

### Photo-thrombosis in freely moving rats

Focal ischemia in freely-moving rats was induced by using the modified photo-thrombosis, which was similar to the previous study. Three laser wavelengths (473 nm, 15–20 mW or 593 nm, 20–0 mW) or LED (565 nm, 5–10 mW) irradiation were explored by delivering into the hippocampus and/or amygdala using a 200-μm diameter optic fiber through the implanted cannulas. Animals received a 30-min illumination at home cage 1 h after Rose Bengal solution injection (100 mg/kg, i.p.; Sigma) or vehicle (saline, 10 ml/kg).

### TTC staining

2,3,5-triphenyl tetrazolium chloride (TTC; Sigma) solution (1%), which was dissolved in artificial CSF (ACSF) solution, was used. Rats were killed under pentobarbital anesthesia after experiments. Brain sections (400 μm in thickness) were obtained with a microtome (VT1000, Leica) and were immediately immersed into the TTC solution at 37°C for 15 min. TTC was metabolized to formazan by the dehydrogenase so that health tissues were stained as red while left the infarct area as white. Sections were transferred into the 4% paraformaldehyde (PFA) for fixation overnight and then mounted onto coverslips and photographed with a digital camera. The injury regions were analyzed using the ImageJ, which was represented by the percentage of the infarct area relative to the whole brain slice.

### Immunohistochemistry

The animals were anesthetized by pentobarbital sodium injection (80 mg/kg, i.p.; Sigma). Perfusion was performed with 0.01 m PBS followed by 4% PFA. The brain was removed and placed in PFA for posterior fixation. The fixed brain was cut with a vibratome (Leica VT1000S) in 50 μm. After washing (0.01 m PBS, 10 min for three times), brain sections were permeabilized and blocked by using 0.3% Triton X-100 and 5% BSA in PBS for 1 h at room temperature (RT). Then sections were incubated with primary antibodies of GFAP (chicken, 1:1000) overnight at 4°C in a humidified chamber. After being washed (0.01 m PBS, 10 min for three times), primary antibodies were visualized separately with secondary antibodies including antibody to chicken Alexa Fluor 488 for GFAP. After staining, sections were mounted onto gelatin-coated glass slides and then counterstained with neutral resin. Images were taken under confocal microscope (Olympus FV3000).

### Nissl staining

Sections at 50-μm thickness were obtained by using a vibratome and mounted onto gelatin-coated glass slides. After rinsed 1 min in ddH_2_O, the slides were dipped in 1% sulfur violet dye solution for 5 min. Then, the slides were rinsed twice in changed ddH_2_O for 5 min each time. Dehydrated the slides by dipping in following solutions in turn: 75% ethanol, 90% ethanol, 95% ethanol, and twice in changed 100% ethanol solutions, 2 min for each time. Finally, the slides were dipped twice in changed 100% xylene solutions for 5 min each time. The sections were counterstained with neutral resin and images were taken under microscope.

### Contextual fear conditioning

Rats were placed in the box (MED Associates) and allowed to freely explore for 2 min before receiving foot shocks (0.8 mA, 2 s, five trials) with 2-min intervals. To test contextual fear memory, rats were placed into the conditioned context for 5 min without foot shocks at different time points after training. The movie and freezing time were automatically recorded during all processes by the software to represent the level of learning and memory.

For measuring the pain sensitivity, rats were placed in the same box used for fear conditioning without habituation. Foot shock was delivered, and the intensity was increased start from 0.1 mA with the stepwise of 0.05 mA (200-ms duration, 20-s interval) until the electric intensity induced the first jump-flinch which was recorded as the pain threshold for this animal.

### Elevated plus maze (EPM) test

The rats were placed individually on the central platform of the EPM (Med Associates) facing a fixed open arm. The EPM consisted of two open arms (52 × 11.5 cm) and two closed arms (52 × 11.5 × 42 cm) and was elevated 74 cm above the floor. The rats were allowed to freely explore open arms and closed arms for 5 min, the behaviors were recorded using a vertically mounted video camera linked to a monitor in an adjacent room. The time spent in the open arms and the total time of moving was calculated.

### Viral tracing

RV-dG-GFP/dsRed (∼3 × 10^8^ tu/ml), the glycoprotein-deficient rabies virus as a retrograde tool was used to verify the projection between the hippocampus and amygdala. The surgery of mice was conducted under pentobarbital anesthesia with pentobarbital anesthesia (60 mg/kg, i.p.). Virus was stereotaxically injected into the unilateral hippocampus or amygdala (hippocampus: AP = −2.4 mm, ML = 2.9 mm, DV = –2.75 mm; amygdala: AP = –2.05 mm, ML = 2.87 mm, DV = –4.65 mm, from the bregma) of each mouse through pulled glass pipettes using the Nanoliter 2000 system (WPI) at the speed of 0.1 μl/min. Nine days later, animals were anesthetized and perfused with 4% PFA. Brains were postfixed in 4% PFA overnight, immersed in 30% sucrose in PBS, and cut into 40-μm sections on a microtome for the DAPI staining and confocal scanning (FV1000, Olympus).

### Slice electrophysiological recording

Basal synaptic transmission in the hippocampus or amygdala was recorded at 5 h or 1 d after focal ischemia induction. The slice preparation protocol was similar to previous studies. Briefly, rats were anesthetized by diethyl ether and decollated. Brains were dissected into the ice-cold ACSF (pH 7.2–7.4, bubbled with 95% O_2_ + 5% CO_2_, osmolarity, 290–300 mOsm kg^−1^). Coronal brain sections (350 μm) were obtained by a microtome (VT1000, Leica) and transferred into an incubation chamber with 37°C for 40 min, and then brain slices were maintained at RT. Borosilicate electrodes (3–5 M) were used, the field EPSPs (fEPSP) were recorded either from the hippocampal CA1 (Schaffer-CA1 pathway) or the basal lateral amygdala (cortical-amygdala pathway) using a glass micropipette (3–6 MΩ) filled with ACSF. The input-output (I/O) curve and paired-pulse ratio (PPR) were examined. Data acquisition and analysis were performed by a Multiclamp 700B/Digidata 1440A system (Molecular Devices Inc.). For each slice, the I/O curve was conducted before PPR to detect the basic excitability of the pathways (Mereu et al., 2000). Stimulus intensity was increased from 0 to 200 μA (stepwise 20 μA) for the Schaffer-CA1 recording and increased from 0 to 500 μA (stepwise 50 μA) for cortical-amygdala recording. An I/O curve was constructed by stimulus intensity against the evoked fEPSP. Two consecutive stimuli with paired pulses were given with different interstimulus intervals (ISIs: 50, 100, 150, 200 ms) to test the PPR, which was calculated by the ratio of the fEPSP amplitude from the second stimulus versus to the first stimulus.

### Analysis

Statistical analysis was performed by SPSS. Student’s *t* test was used to analyze the difference of memory test, and the repeated measure was used to analyze the difference of learning curve and the basal synaptic transmission of brain slice recording, including the I/O curve and PPR ratio. One-way ANOVA was used to compare ischemic size across time windows. The significance used was *p* < 0.05.

## Results

### Focal ischemia induction in freely moving rats

**Figure 1.**
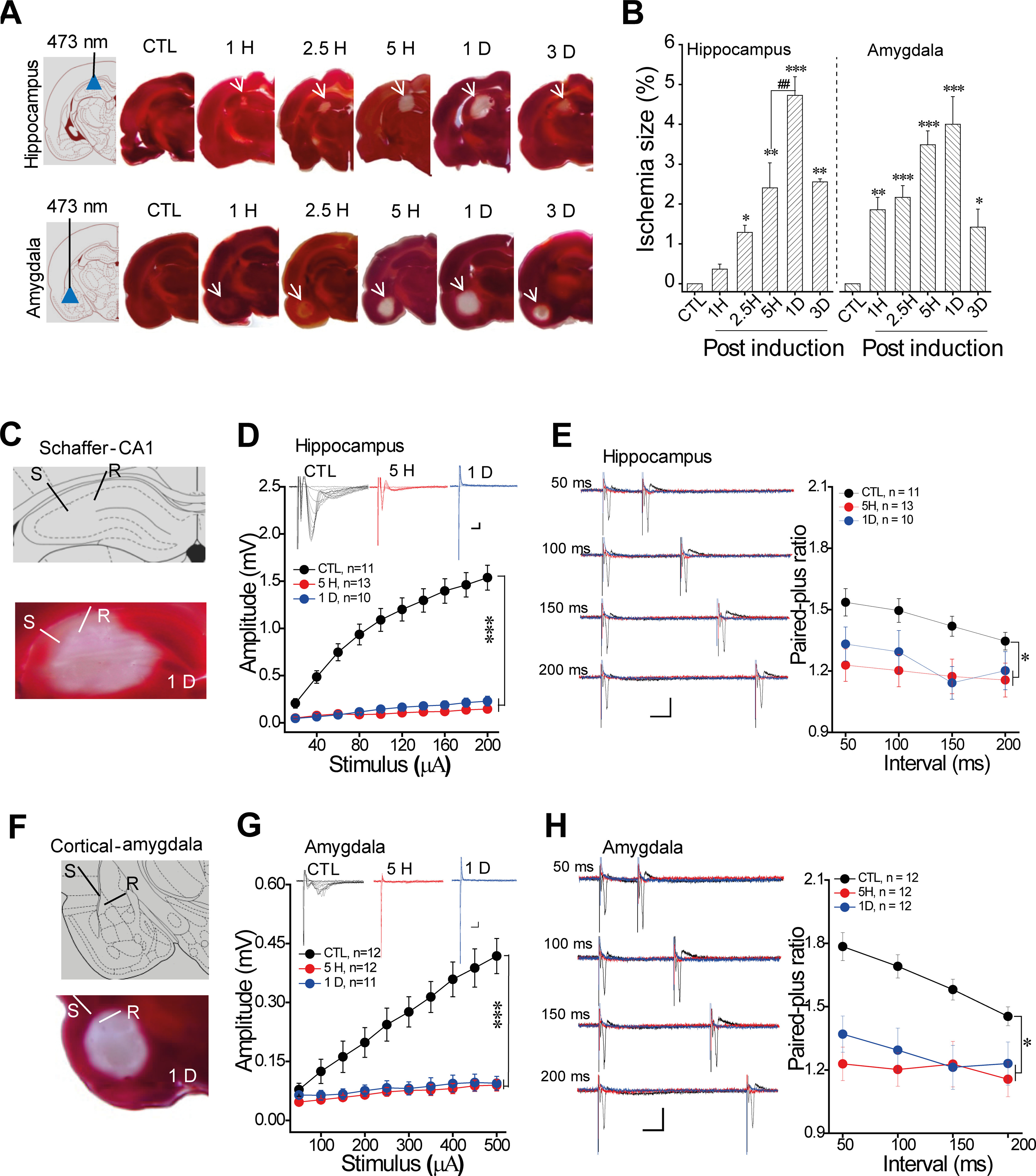
Photo-thrombosis induced focal ischemia in freely moving rats. ***A***, Schematic and the representation of focal ischemia injury in unilateral hippocampus or amygdala by TTC staining with different time windows (from 1 h to 3 d), the red area was the normal tissue and the arrow showed the white ischemic area. ***B***, Group data showed the developed ischemia injury after 473-nm laser illumination. ***C***, Schaffer-CA1 pathway recording with focal ischemia injury: S = the stimulus site, R = the recording site. ***D***, Representative traces and the group data of I/O curve, and (***E***) the traces (left) and group data of PPR from the recording in the Schaffer-CA1 pathway. ***F***, Cortical-amygdala pathway recording with focal ischemia injury. ***G***, The traces and group data of I/O curve, and (***H***) the traces (Left) and group data of PPR from the recording in cortical-amygdala pathway. Mean ± SEM for each bar. Statistics were performed using one-way ANOVA (***B***) and repeated ANOVA (***D–H***). Scale in traces: 2 ms, *x*-axis = 2 ms, *y*-axis = 0.2 mV; PPR: *x*-axis = 25 ms, *y*-axis = 0.2 mV; *p < 0.05, **p < 0.01, ***p < 0.001, compared with control groups, ^##^*p* < 0.01 used for the difference between the 5H and 1D. Each slice was 400 μm. Extended information illustrating the effects of conditions on ischemic induction is available in Extended Data Figure 1-1, the trace of TTC staining from a single rat across the injury site with 1-d ischemia is available in Extended Data Figure 1-2 and the Nissl and anti-GFAP staining at day 7 after focal ischemia in Extended Data Figure 1-3.

Most of the previous animal studies conducted ischemic damage under anesthesia that may compromise the evaluation of subsequent behavioral cognitions. Here, we modified the method of photo-thrombosis in freely moving rats (Fig. 1*A*). The 473-nm laser illumination was optimal for ischemia induction as shown by TTC staining. The slice with the largest size of damage in a series of sections was used to quantify the ischemia area because it was nicely correlated to the injury volume calculated by all areas of the sections. TTC staining showed that the neural injury in the hippocampus was developed as early as 1–2.5 h, reached to the maximal level at 1 d after photo-thrombosis (*F*_(5,12)_ = 21.1, *p* < 0.001; *post hoc*: 1H, *p* = 0.536; 2.5H, *p* = 0.045; 5H, *p* = 0.001; 1D, *p* < 0.001; 3D, *p* = 0.001; compared with Control (CTL); 5H vs 1D, *p* = 0.001; Fig. 1*A*,*B*). Results from the amygdala were similar with a slightly faster development as the neural injury was significant as early as 1 h and reached to the maximal level at 5 h after photo-thrombosis (*F*_(5,15)_ = 8.9, *p* < 0.001; *post hoc*: 1H, *p* = 0.018; 2.5H, *p* = 0.005; 5H, *p* < 0.001; 1D, *p* < 0.001; 3D, *p* = 0.037; 5H vs 1D, *p* = 0.474; Fig. 1*A*,*B*). We further examined the I/O curve and PPR in the brain slices recording, which is useful for evaluating neural injury on basal transmission after ischemia induction (Lee et al., 2014). The results showed that ischemia induction dramatically impaired the basal synaptic transmission in Schaffer-CA1 (I/O curve: hippocampus: *F*_(2,31)_ = 70, *p* < 0.001; 5H, *p* < 0.001; 1D, *p* < 0.001; compared with CTL; 5H vs 1D, *p* = 0.644; PPR: hippocampus, *F*_(2,31)_ = 3.728, *p* = 0.035; *post hoc*: 5H, *p* = 0.013; 1D, *p* = 0.048; compared with CTL; 5H vs 1D, *p* = 0.608; Fig. 1*C–E*) and cortical-amygdala pathways (I/O curve: *F*_(4,55)_ = 33.6, *p* < 0.001; *post hoc*: 5H and 1D, both *p* < 0.001, compared with CTL; 5H vs 1D, *p* = 0.77; PPR: *F*_(2,33)_ = 6.25, *p* = 0.0027; *post hoc*: 5H, *p* = 0.012; 1D, *p* = 0.025; compared with CTL; 5H vs 1D, *p* = 0.698; Fig. 1*F–H*). Thus, photo-thrombosis induced focal ischemic injury was a developing process, the injury area was maximum at around 1 d later and the basal synaptic transmission was blocked at 5 h.

10.1523/ENEURO.0398-20.2021.f1-1 Extended Data Figure 1-1 Focal ischemia induction with different conditions in freely moving rats. ***A***, Three conditions. Left, 30-min irradiation with 593-nm laser. Middle, 30-min irradiation with 565-nm LED. Right, 30-min irradiation with 473-nm laser. ***B***, ***C***, The TTC staining 1 d after irradiation in the hippocampus or amygdala. Each slice was 400 μm. Download Figure 1-1, TIF file.

**Figure 2.**
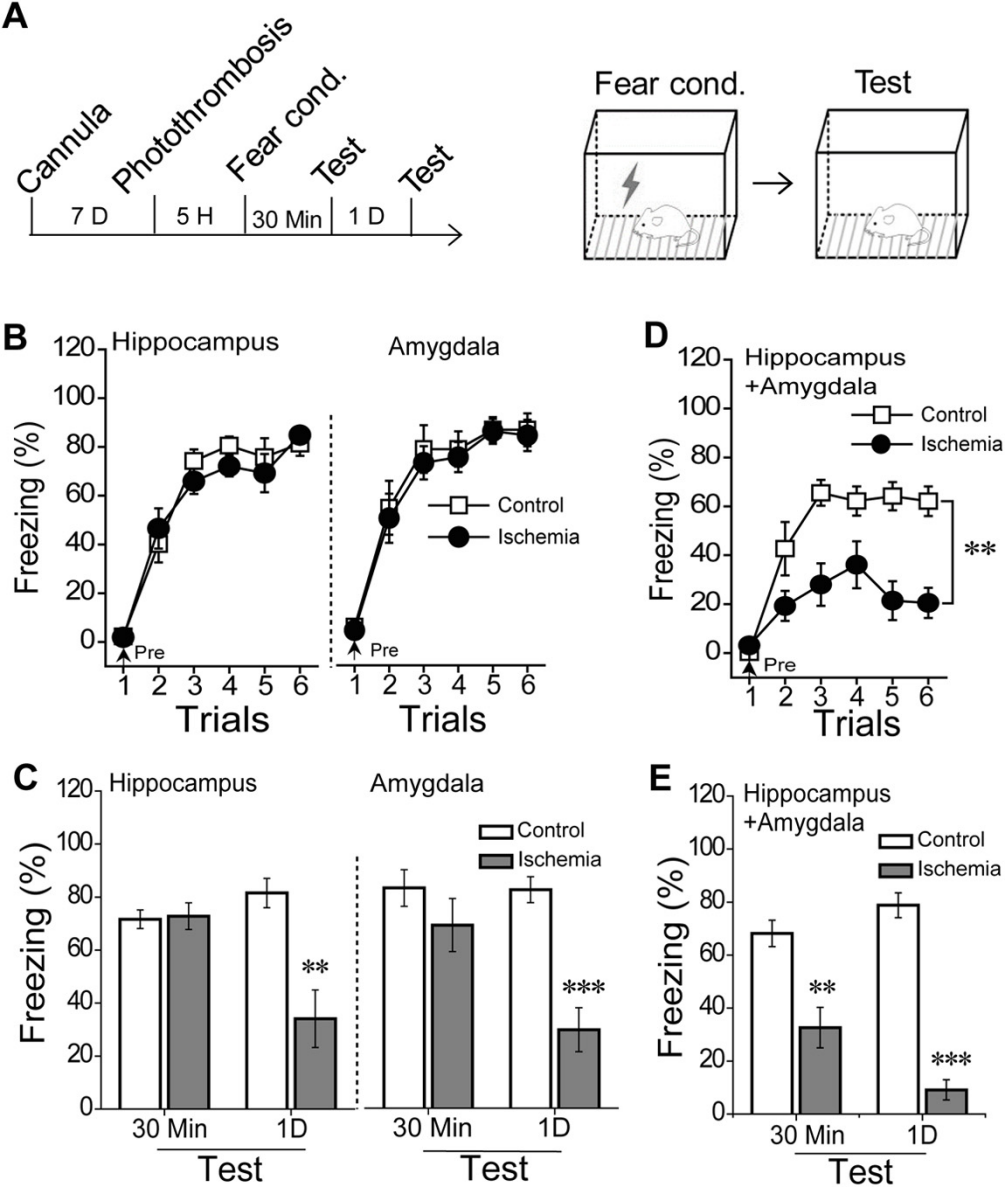
Fear memory formation with 5-h ischemia injury in hippocampus or/and amygdala. ***A***, Rats received an ischemia induction 5 h before training, and the schematic of fear conditioning. ***B***, Learning curve during fear conditioning with 5-h ischemic injury in the bilateral hippocampus (left, control, *n* = 11; ischemia, *n* = 11) or bilateral amygdala (right, control, *n* = 8; ischemia, *n* = 9) and (***C***) dependent contextual 30-min STM and 1-d LTM. ***D***, The learning curve from the rats with 5-h ischemia in both hippocampus and amygdala before training and (***E***) the dependent contextual 30-min STM and 1-d LTM (control, *n* = 8; ischemia, *n* = 8). Mean ± SEM for each bar, statistics were performed using repeated measure ANOVA (***B***) and independent *t* test (***C***); *p < 0.05, **p < 0.01, ***p < 0.001, compared with control groups. Extended information illustrating the effects of Rose Bengal treatment on contextual fear formation is available in Extended Data Figure 2-1, the trace of TTC staining in a single rat with 1-d ischemia in both hippocampus and amygdala is available in Extended Data Figure 2-2, and the effects of 5-h ischemia in both the hippocampus and amygdala on pain threshold, general activity, and anxiety are available in Extended Data Figure 2-3, and the correlation between the injury size of the adjacent areas and the freezing time during the memory test in Extended Data Figure 2-4.

10.1523/ENEURO.0398-20.2021.f1-2 Extended Data Figure 1-2 Brain sections with TTC staining from one animal showed the whole injury site with 1-d ischemia. ***A***, One-day focal ischemia in the unilateral hippocampus. ***B***, One-day focal ischemia in the unilateral amygdala. Each slice was 400 μm. Download Figure 1-2, TIF file.

**Figure 3.**
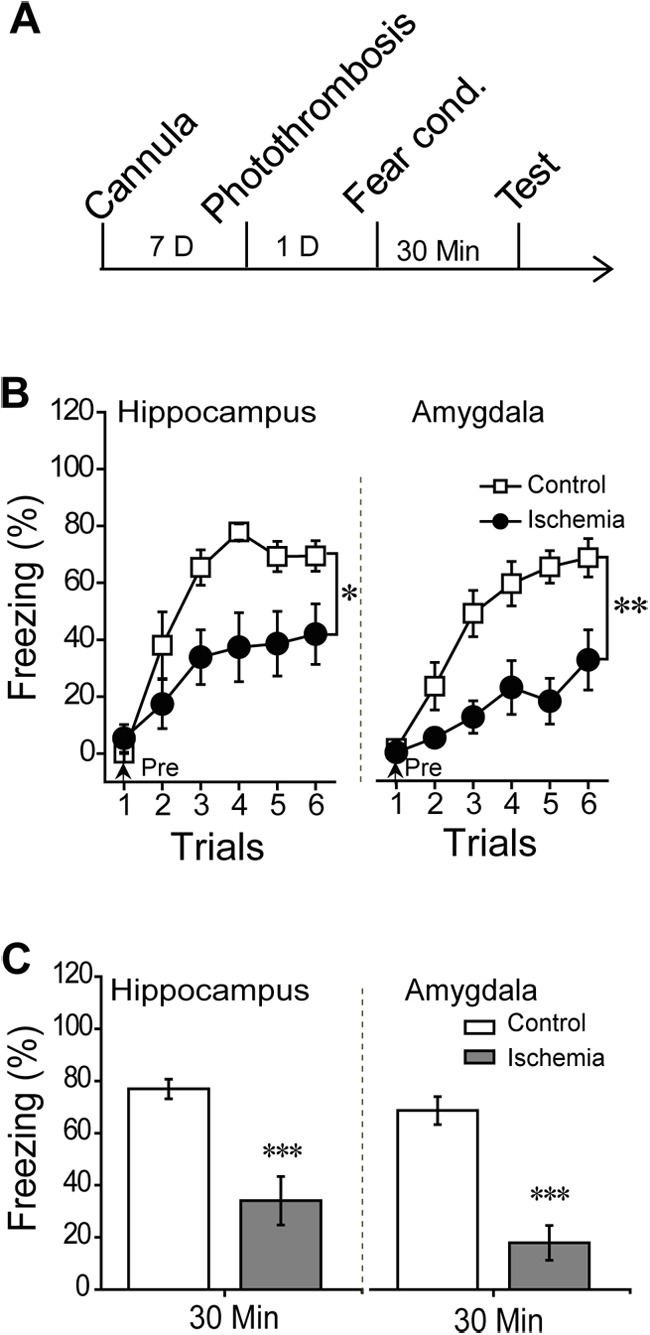
Fear memory acquisition in the rats with 1-d focal ischemia injury in the bilateral hippocampus or bilateral amygdala. ***A***, Rats were conducted fear conditioning following by 1-d ischemia injury. ***B***, Learning curve in the rats with 1-d ischemia injury in the bilateral hippocampus (left: control, *n* = 9; ischemia, *n* = 8) or bilateral amygdala (right: control, *n* = 9; ischemia, *n* = 9). ***C***, Dependent 30-min STM test. Mean ± SEM for each bar. Statistics were performed using repeated measure ANOVA (***B***) or independent *t* test (***C***); ***p < 0.001, compared with control groups.

10.1523/ENEURO.0398-20.2021.f1-3 Extended Data Figure 1-3 The sustained damage at day 7 after focal ischemia was represented by (***A***) 7-d Nissl staining and (***B***) the formation of glial scar indicated by anti-GFAP staining. Each slice was 50 μm. Download Figure 1-3, TIF file.

### The ischemic injury occurred in the hippocampus or/and amygdala for memory acquisition

To examine the effect of focal ischemia injury on the fear memory process, rats with cannula implantation were received fear conditioning at 5 h after induction (Fig. 2*A*). Rats with focal ischemia in the hippocampus or amygdala showed same the learning curve to controls (hippocampus, *F*_(1,20)_ = 0.17, *p* = 0.683; amygdala, *F*_(1,15)_ = 0.13, *p* = 0.722; repeated measure, Fig. 2*B*), as well as 30-min short-term memory (STM; hippocampus, *p* = 0.856; amygdala, *p* = 0.308; *t* test; Fig. 2*C*). Nevertheless, these rats displayed amnesia for long-term memory (LTM) as the freezing levels in the memory retrieval test 1 d after photo-thrombosis (hippocampus: *p* = 0.004; amygdala: *p* < 0.001; *t* test; Fig. 2*C*). Rose Bengal treatment alone had no effects on conditioning learning, STM, and LTM. We hypothesized that the fear memory acquisition could be processed by the intact hippocampus if the amygdala was impaired, and vice versa, resulting in unaffected learning and STM under the condition of 5-h ischemic injury. To clarify this possibility, we induced ischemic damage in both the hippocampus and amygdala and then subjected the rats to contextual fear conditioning at 5 h after photo-thrombosis. Learning curve, 30-min STM, and 1-d LTM were all impaired as compared with Rose Bengal controls (learning curve, *F*_(1,14)_ = 19.2, *p* < 0.001, repeated measure; 30 min, *p* = 0.002; 1D, *p* < 0.001, *t* test; Fig. 2*D*,*E*). We further confirmed the changed freezing behavior was because of the memory effects, as the 5-h ischemia both in the hippocampus and amygdala did not affect the pain threshold, anxiety, and movements. Thus, either the hippocampus or the amygdala alone could acquire the fear learning successfully. While the acquisition process was impaired when the ischemia was applied to both the hippocampus and amygdala. These findings suggested that the complementary roles between the hippocampus and the amygdala during memory acquisition, which could be blocked by the injury in both sites.

10.1523/ENEURO.0398-20.2021.f2-1 Extended Data Figure 2-1 The effects of Rose Bengal treatment on contextual fear formation. ***A***, Schematic, rats were injected Rose Bengal (100 mg/kg, i.p.) 1 h before conditioning, and saline (10 ml/kg, i.p.) as control. ***B***, Learning curve during conditioning (repeated measure ANOVA: *F*_(1,8)_ = 0.08, *p* = 0.776). ***C***, 30-min and 1-d dependent contextual tests (*t* test: 30 min, *p* = 0.156; 1 d, *p* = 0.725). Control, *n* = 5, Rose Bengal, *n* = 5. Mean ± SEM for each bar. Download Figure 2-1, TIF file.

10.1523/ENEURO.0398-20.2021.f2-2 Extended Data Figure 2-2 Brain sections with TTC staining from a single rat one with 1-d ischemia in both hippocampus and amygdala. Each slice was 400 μm. Download Figure 2-2, TIF file.

10.1523/ENEURO.0398-20.2021.f2-3 Extended Data Figure 2-3 The effects of 5-h ischemia in both the hippocampus and amygdala on other behaviors. ***A***, Pain threshold test in fear conditioning box (control, *n* = 10 rats, ischemia, *n* = 9 rats; T = 2, *p* = 0.062, *t* test). ***B***, Total time moving during EPM test (control, *n* = 10, ischemia, *n* = 9; T = 0.679, *p* = 0.502, *t* test). ***C***, The percentage of time spend in open arms during EPM test (control, *n* = 10, ischemia, *n* = 9; T = 0.846, *p* = 0.409, *t* test). Mean ± SEM for each bar. Download Figure 2-3, TIF file.

**Figure 4.**
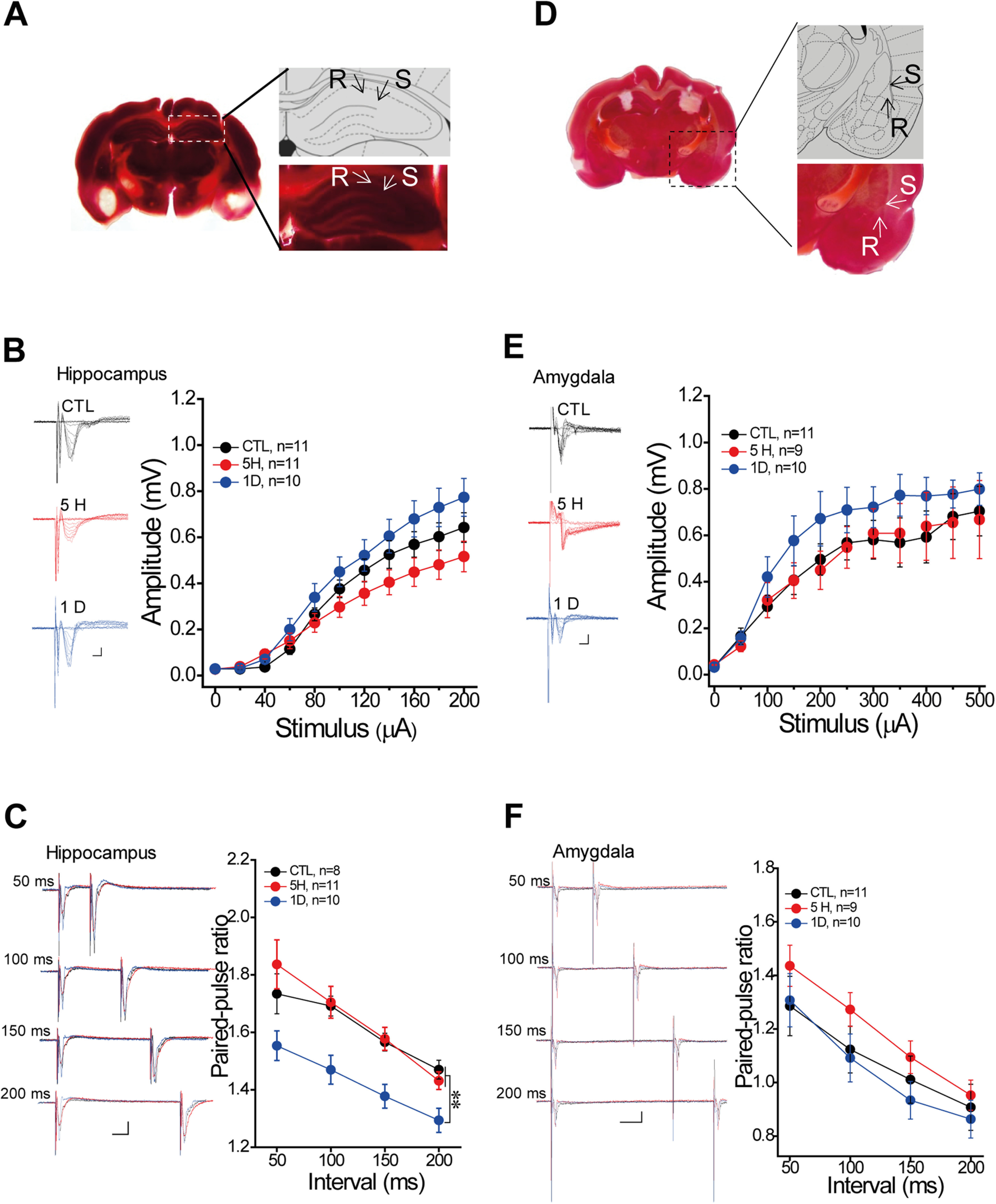
The basal transmission in the remote site after focal ischemia induction. ***A***, Schaffer-CA1 recording with bilateral amygdala ischemia: S = the stimulus site, R = the recording site. ***B***, Representative traces (left) and group data of I/O curve, and (***C***) the traces (left) and group data of PPR from hippocampal recording under the ischemia of the bilateral amygdala. ***D***, Cortical-amygdala pathway with bilateral hippocampus ischemia. ***E***, The traces (left) and group data of I/O curve, and (***F***) the traces (left) and group data of PPR from amygdalar recording under the ischemia of bilateral hippocampus (the left: trace). Control, control groups with surgery and Rose Bengal; 5 H, 5 h; 1 D, 1 d after photo-thrombosis. Mean ± SEM for each bar. Scale in trace: 2 ms, *x*-axis = 2 ms, *y*-axis = 0.2 mV; PPR: *x*-axis = 25 ms, *y*-axis = 0.2 mV. Statistics were performed using repeated measure ANOVA with *post hoc* test, **p < 0.01, compared with control groups. Extended information illustrating the interconnection between the hippocampus and amygdala using non-transsynaptic retrograde rabies virus tracing is available in Extended Data Figure 4-1.

10.1523/ENEURO.0398-20.2021.f2-4 Extended Data Figure 2-4 The effects of the injury size at adjacent areas on the freezing behaviors. ***A***, The white dash line indicated the damage to the adjacent areas out of the region of interest (ROI). Each slice was 400 μm. There was no correlation between the injury size of adjacent areas and the freezing time during the (***B***) 30-min (*r* = 0.003, *p* = 0.45) and (***C***) 1-d (*r* = 0.22, *p* = 0.57) memory test (*n* = 4). ***D***, ***E***, One rat with focal ischemia at the out of the ROI (the arrow) showed the same fear acquisition ability to its literature control. Download Figure 2-4, TIF file.

### Focal ischemia produced remote changes of synaptic transmission in a time-dependent manner

Interestingly, the memory acquisition under focal ischemia in the hippocampus or amygdala alone was blocked when the ischemic injury was extended to 1 d before conditioning (Fig. 3*A*). The animals with 1-d ischemia in the hippocampus or amygdala significantly impaired learning curve (hippocampus *F*_(1,15)_ = 7.66, *p* = 0.014; amygdala, *F*_(1,16)_ = 13.3, *p* = 0.002, repeated measure; Fig. 3*B*) and 30-min STM (hippocampus, *p* < 0.001; amygdala, *p* < 0.001, *t* test; Fig. 3*C*), as previous reports have demonstrated reciprocal projections between the hippocampus and amygdala (Pitkänen et al., 2000; Kishi et al., 2006), which was further confirmed by the retrograde tracing virus in this study (Extended Data Fig. 4-1). Thus, we supposed that the size with neural injury induced by the ischemia at 1 d was much greater than those at 5 h after photo-thrombosis, which resulted in the loss function of compensatory pathways. To address this hypothesis, we induced focal ischemia in the hippocampus or amygdala and examined the basal transmission in the cortical-amygdala or Schaffer-CA1 pathway, respectively (Fig. 4*A*,*D*). We found that focal ischemia in the amygdala did not affect the synaptic transmission in the Schaffer-CA1 pathway which was indicated by an unchanged I/O curve at 5 h and 1 d after photo-thrombosis (*F*_(2,26)_ = 2.39, *p* = 0.25, repeated measure; Fig. 4*B*). However, the focal ischemia in the amygdala indeed reduced the PPR of the Schaffer-CA1 pathway at 1 d but not 5 h after photo-thrombosis (*F*_(2,26)_ = 7.92, *p* = 0.002, repeated measure; *post hoc*: 5H, *p* = 0.725; 1D, *p* = 0.005, compared with control; Fig. 4*C*). This result indicated that focal ischemic injury in the amygdala could change the synaptic transmission of both amygdala and hippocampus at 1 d after photo-thrombosis, suggesting a functional interaction between the amygdala and hippocampus (Richter-Levin and Akirav, 2000). While, ischemia in the hippocampus, had no effect on the synaptic transmission in the cortex-amygdala pathway as it showed intact I/O curve and PPR in either 5 h or 1 d after photo-thrombosis (I/O curve: *F*_(2,27)_ = 0.859, *p* = 0.434; PPR: *F*_(2,27)_ = 0.834, *p* = 0.444, repeated measure; Fig. 4*E*,*F*). It implicates that amygdalar ischemia could lead to more severe damage in this paradigm than hippocampal injury did because the former resulted in functional impairments of the both at 1 d after photo-thrombosis.

10.1523/ENEURO.0398-20.2021.f4-1 Extended Data Figure 4-1 The retrograde tracing of rabies virus in hippocampus or amygdala. ***A***, Schematic: a non-transsynaptic rabies virus carried the GFP (RV-dg-GFP) as the reporter was injected into the unilateral hippocampus and a rabies virus carried the Dsred (RV-dg-dsRed) as the reporter was injected into the unilateral amygdala. ***B***, ***C***, The dsRed expression in the local injection site of the amygdala. ***D***, ***E***, The dsRed-positive cells expressed in the hippocampus. ***F***, The GFP expression in the local injection site of the hippocampus. ***G***, the GFP-positive cells expressed in the amygdala. Amy, amygdala; Hip, hippocampus. Each slice was 40 μm, confocal microscope scanning. All scale bars were 100 μm. Download Figure 4-1, TIF file.

### Memory was acquired via relearning in the rats with ischemia-induced retrieval impairments

**Figure 5.**
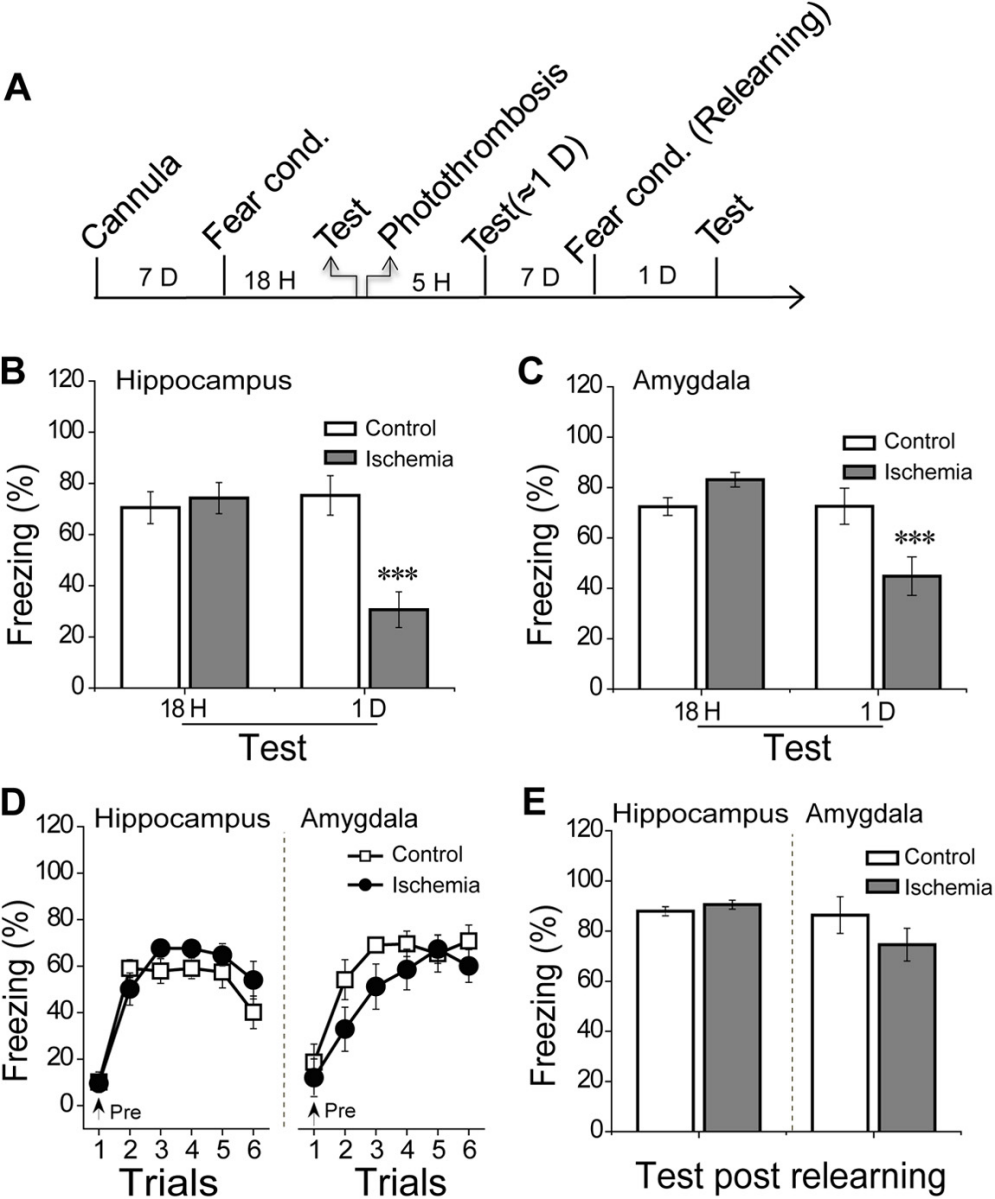
The effects of focal ischemia on memory retrieval and relearning. ***A***, Schematic: rats received fear conditioning and dependent 18-h memory test and then given photo-thrombosis 5 h before the 1-d test, and the rats received a relearning process 7 d later. ***B***, ***C***, 18-h and 1-d dependent contextual tests, rats were given the photo-thrombosis in the bilateral hippocampus (control, *n* = 8, ischemia, *n* = 9) or bilateral amygdala (control, *n* = 9, ischemia, *n* = 10) after 18-h test immediately. ***D***, ***E***, Relearning curve 7 d after photo-thrombosis and dependent 1-d contextual test: (left) rats with ischemia in the bilateral hippocampus (control, *n* = 8, ischemia, *n* = 8) and (right) rats with ischemia in the bilateral amygdala (control, *n* = 9, ischemia, *n* = 9). Repeated measure with *post hoc* test was used in the (***D***) and Student’s *t* test was in (***B***, ***C***, ***E***) to analyze the difference. Each bar represents mean ± SEM; ***p < 0.001, compared with control groups.

The impaired 1-d memory test indicated that both the hippocampus and amygdala were critical for memory retrieval. To confirm this question, animals were received 5-h focal ischemia before the 1-d memory test (Fig. 5*A*). An 18-h test before photo-thrombosis was used to prove these animals have the same baseline of LTM (hippocampus, *p* = 0.98; amygdala, *p* = 0.97; Fig. 5*B*,*C*, left). Similarly, the 5-h focal ischemia either in the hippocampus or amygdala was impaired the following 1-d LTM (hippocampus, T = 4.19, *p* < 0.001; amygdala, T = 4.07, *p* < 0.001; Fig. 5*B*,*C*, right), suggesting that 5-h ischemia was sufficient to impair LTM retrieval. However, this ischemic injury did not block the animals in relearning of contextual fear 7 d after photo-thrombosis and rememorizing 1 d after relearning (learning curve: hippocampus, *F*_(1,14)_ = 1.57, *p* = 0.22; amygdala, *F*_(1,16)_ = 1.82, *p* = 0.19, repeated measure; relearning test: hippocampus, *p* = 0.48; amygdala, *p* = 0.27, *t* test; Fig. 5*D*). Thus, the compensatory roles between the hippocampus and amygdala were also suitable for the relearning process.

## Discussion

We used a modified photo-thrombosis that targeted the hippocampus and amygdala in freely moving rats and found a compensatory rule one for the another during contextual fear acquisition. The results showed that the animals with 5-h ischemia in the hippocampus or amygdala alone could fulfill fear memory acquisition, while still showed impaired 1-d memory retention. In contrast, memory acquisition was blocked when focal ischemia was applied to both the hippocampus and the amygdala. These findings suggest that either the hippocampus or the amygdala alone is sufficient for the associative memory acquisition but not for LTM formation, implicating their complementary roles in this process.

The different roles of the hippocampus and amygdala in the stages of contextual fear memory are still elusive. The previous studies with pharmacological inactivation or lesion show that fear memory can be acquired when some brain regions are impaired, it suggests the existence of primary brain regions-dependent and compensatory regions independent (Wiltgen et al., 2006; Poulos et al., 2010; Zelikowsky et al., 2012; Zhou et al., 2016). However, the acute pharmacological inactivation or permanent lesion could not provide an appropriate time window for observation. In this study, we employed a modified the model of photo-thrombosis to address this question with three major reasons. First, stroke often induced cognitive impairments in the clinic no matter where the site of the ischemia is (Esiri et al., 1999), and the hippocampus and amygdala are susceptible in this type of injury (Sachdev et al., 2007; Jagust et al., 2008; Carty et al., 2010; Lin et al., 2014). Second, they are likely improved either spontaneously or through rehabilitation in some patients with ischemia attacks (Snaphaan and de Leeuw, 2007). Third, the damage induced by ischemia shows a developing process that it enables us to test the impact of different levels of damage on contextual fear memory, which is ignored in the previous (Felberg et al., 2000). This model could be also applied to study the mechanism of ischemia induced amnesia and the efficacy of neuroprotective drugs (Esiri et al., 1999; Yu et al., 2015; Jiao et al., 2017).

Although the imaging quality of TTC staining was similar to other studies (Chou et al., 2004; Barth and Mody, 2011; Nishimura et al., 2016), an unsmooth or leaked light spot might induce tiny damage to the adjacent areas in this paradigm which was represented by the TTC staining from four rats, but there was no correlation between the injury size of adjacent areas and the freezing time. We hypothesized that the adjacent areas might be ischemic penumbra which has partial transmembrane potential, or these areas were not critical for contextual memory or network compensation. We preferred the latter as there was one rat with focal ischemia at the out of the region of interest that showed the same fear acquisition ability to its literature control. So, the limited adjacent injury was not related to the behavioral changes in this study. However, for other purposes, the researchers need to adjust the related parameters including the laser power and the location of the optical-fiber tips to reduce the potential damage at adjacent areas. Meanwhile, it was worthwhile to note that the Nissl staining or immunofluorescence is much greater in the high-magnification image than TTC staining (Jiao et al., 2017). We still applied the TTC staining for most experiments because the Nissl staining was not sensitive at the early stage of ischemia, and the tissues were two fragile during 50-μm slice preparation especially for the animals with 24-h injury at the amygdala, and the TTC staining was the good selection for the brain slices that already were applied to the electrophysiological recording. While, the Nissl staining and immunofluorescence were more reliable to provide detailed information about the cellular damage after the formation of the glial scar (such as 7-d ischemia) which may mask the damage when using the TTC staining, as it depends on the dehydrogenases, which are most abundant in mitochondria (Benedek et al., 2006).

The ischemic injury time-dependently affected the fear memory. The 5-h ischemia either in the hippocampus or the amygdala was not sufficient to block memory acquisition. However, the ischemia that happened in both the hippocampus and the amygdala impaired this process. Similarly, when the ischemic time was extended to 1 d, the damage of either the hippocampus or the amygdala blocked the process, as there is a bidirectional projection between the hippocampus and the amygdala (Pitkänen et al., 2000; Kishi et al., 2006), and responsible for contextual fear (Richter-Levin and Akirav, 2000; Seidenbecher et al., 2003). We hypothesized that there is the synergic role of hippocampus and amygdala in memory acquisition, the 5-h ischemia was applied to the hippocampus alone, the compensative amygdala can still fulfill the acquisition process, as although the damage in the hippocampus reached its maximum at 5 h, the synaptic transmission of the amygdala was still intact which was represented by PPR in brain slice recording, and vice versa. If so, the memory acquisition will be blocked when the 5-h ischemia happens in both regions. The 1-d focal ischemia was applied to the hippocampus or amygdala alone also blocked acquisition, which may be explained by the effect of ischemia injury was spread, because the 1 d postfocal ischemia in the amygdala led to the changes in synaptic transmission in the hippocampus as indicated by reduced PPR from brain slice recording. The rats with 7-d ischemia either in the amygdala or hippocampus showed normal relearning and rememorizing, suggesting that the memory system is highly adaptive for relearning or possibly new learning by using the intact parts of the regions. Furthermore, according to the theory of multiple memory systems, some other brain structures also work together with the hippocampus and amygdala to create the entire source of fear memory information (Fendt and Fanselow, 1999; Zelikowsky et al., 2014; Yavas et al., 2019). For example, the coordinated rhythmic (4-Hz) activity between the prelimbic part of prefrontal cortex and amygdala elicits the freezing behaviors (Karalis et al., 2016), the coordinated oscillatory activity between hippocampus and cortex may be responsible for spatially related information to the prelimbic cortex from the hippocampus (Shin and Jadhav, 2016). It is worth investigating the possibility of other brain regions have the compensative role of contextual information when the hippocampus is damaged and has the compensative role of fear behaviors when the amygdala is damaged.

In summary, we developed a whole-scale neurobehavioral evaluation in freely moving rats with focal ischemia to study the interplaying between the hippocampus and the amygdala in memory processing. Both the hippocampus and amygdala were involved in contextual fear memory acquisition, and either of the regions had the compensative effect when the other was impaired. Our findings provided new insight into the circuitry mechanism of contextual fear memory and offered a paradigm for preclinical anti-ischemic drug screening.

## Synthesis

Reviewing Editor: Ayako Watabe, Jikei University School of Medicine Decisions are customarily a result of the Reviewing Editor and the peer reviewers coming together and discussing their recommendations until a consensus is reached. When revisions are invited, a fact-based synthesis statement explaining their decision and outlining what is needed to prepare a revision will be listed below. The following reviewer(s) agreed to reveal their identity: Alexei Morozov, Hiroshi Nomura.

While the authors addressed most of the comments properly, some of the concerns raised by the reviewers are still not well examined.

Major concerns;

The manipulation seems to damage the adjacent areas. However, they only provided some representative images of the focal ischemia injury. In addition, their images are low-magnified ones. The damage of the adjacent areas was not quantified. Therefore, it is impossible to understand what causes the memory impairment.

The authors should provide the data showing the ischemia size of the adjacent regions (e.g. the perirhinal cortex, piriform cortex, striatum, thalamus) and the high magnification images showing cells are damaged or not in these adjacent regions. If their manipulation damaged the adjacent regions, they should add the alternate interpretation of their data and explain the limitation of their method in their discussion.

Minor concerns;

The statement in the abstract “These results suggest that interconnection between the hippocampus and the amygdala is critical for fear memory acquisition” is not very precise because the interconnection has not been manipulated. This statement needs to be changed.
