# Supplementary figures and images for: Developing of Focal Ischemia in the Hippocampus or the Amygdala Reveals a Regional Compensation Rule for Fear Memory Acquisition
Source: eNeuro. 2021 Apr 21;8(2):ENEURO.0398-20.2021. doi: 10.1523/ENEURO.0398-20.2021 (PMC8174052; doi:10.1523/ENEURO.0398-20.2021)

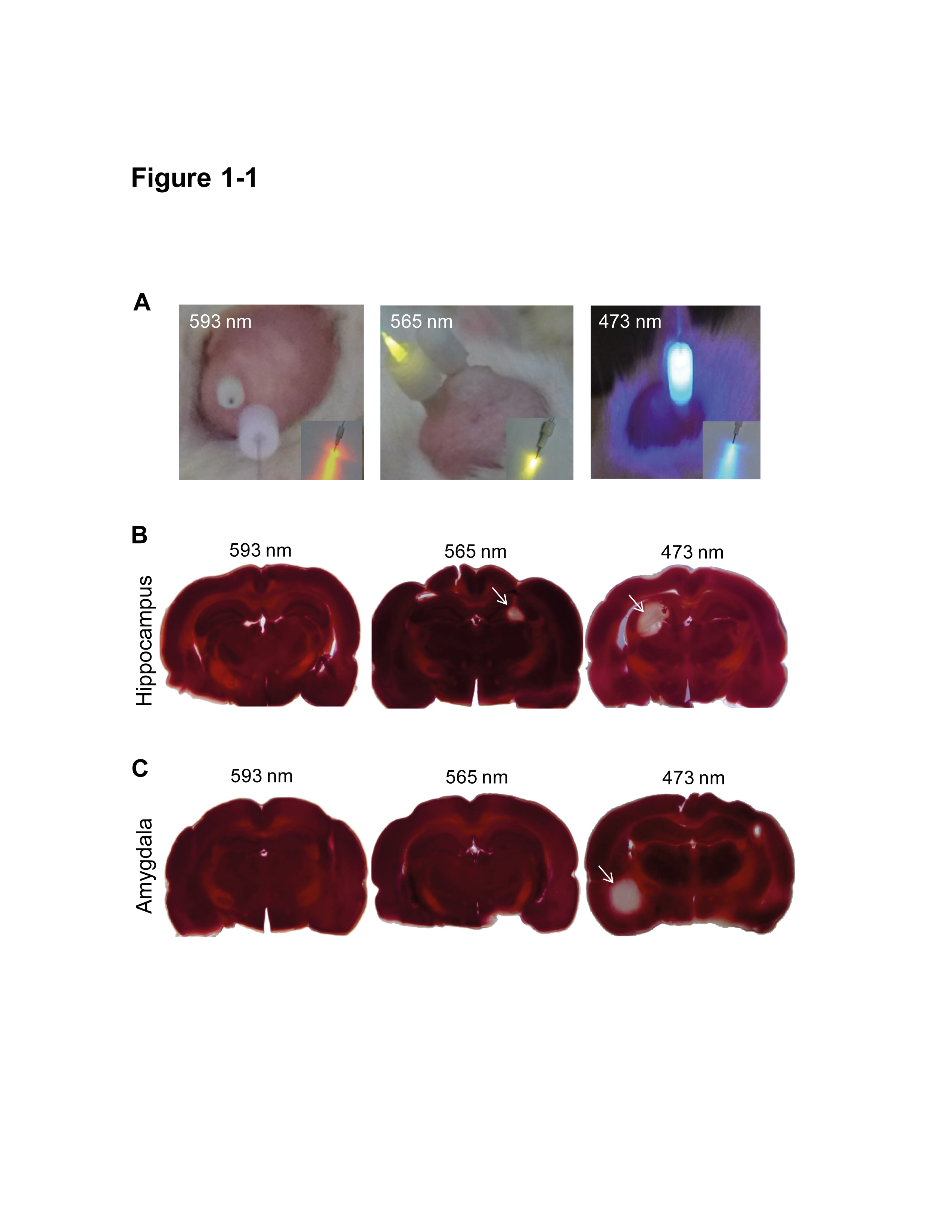

Supplement: Extended Data Figure 1-1 — Focal ischemia induction with different conditions in freely moving rats. A, Three conditions. Left, 30-min irradiation with 593-nm laser. Middle, 30-min irradiation with 565-nm LED. Right, 30-min irradiation with 473-nm laser. B, C, The TTC staining 1 d after irradiation in the hippocampus or amygdala. Each slice was 400 μm. Download Figure 1-1, TIF file. [file enu-eN-NWR-0398-20-s01.tif]

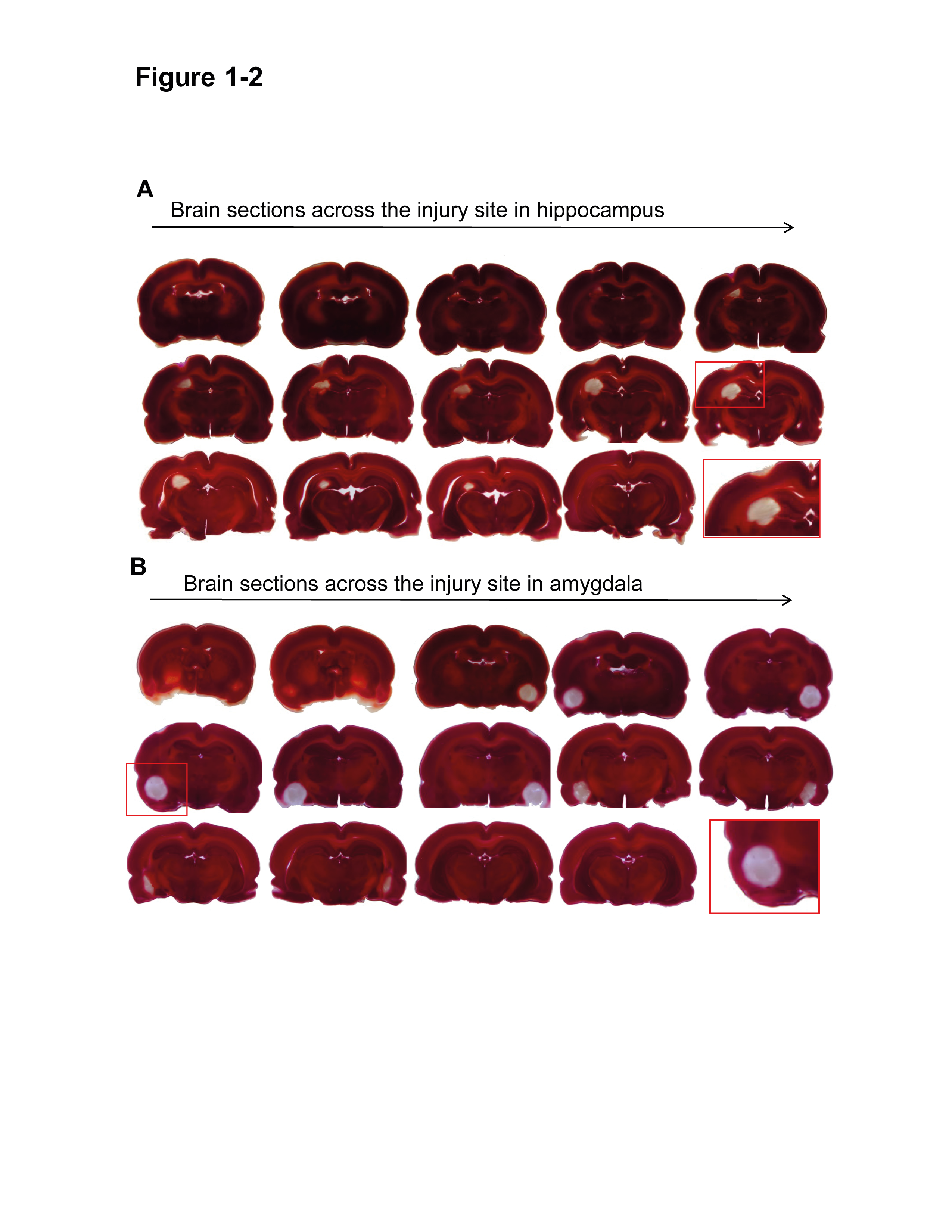

Supplement: Extended Data Figure 1-2 — Brain sections with TTC staining from one animal showed the whole injury site with 1-d ischemia. A, One-day focal ischemia in the unilateral hippocampus. B, One-day focal ischemia in the unilateral amygdala. Each slice was 400 μm. Download Figure 1-2, TIF file. [file enu-eN-NWR-0398-20-s02.tif]

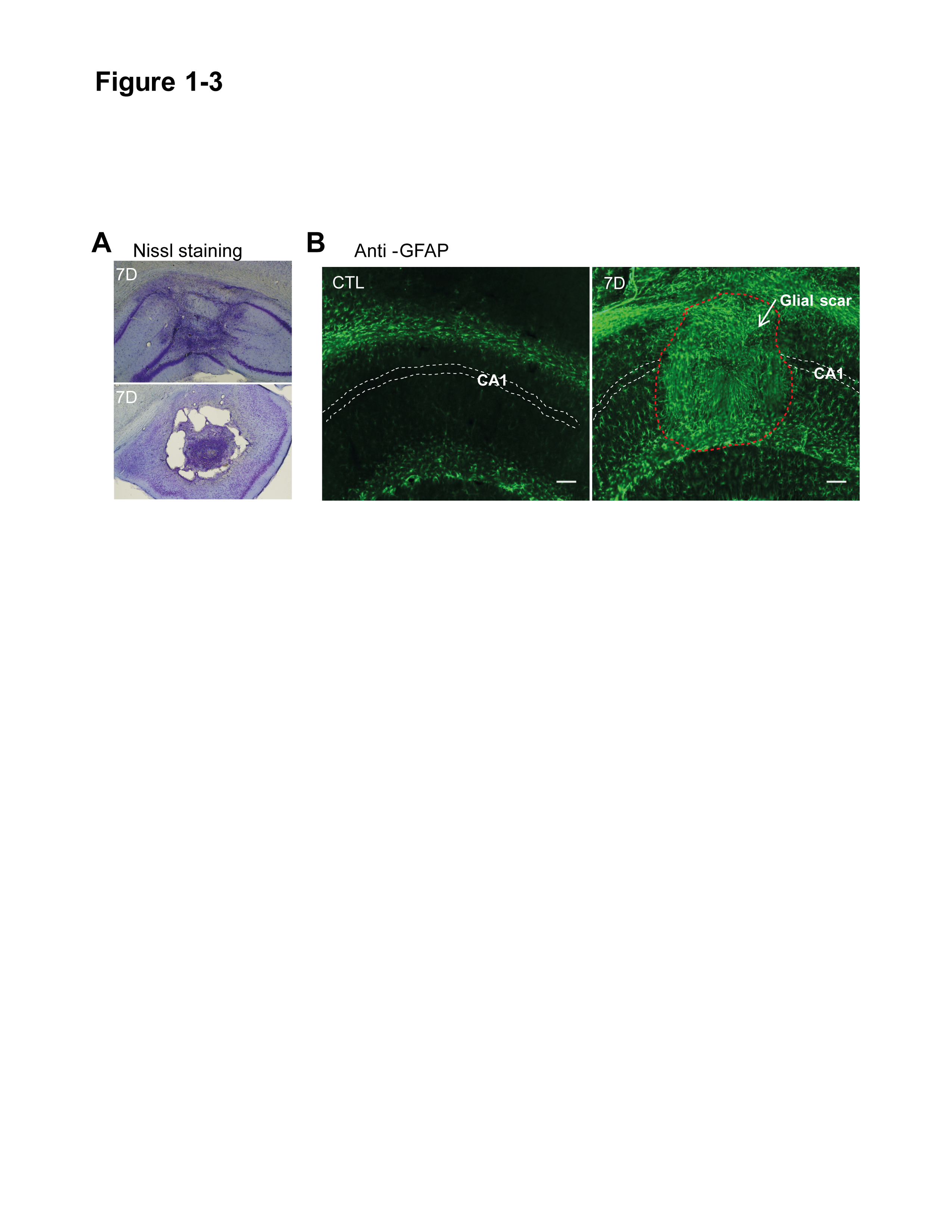

Supplement: Extended Data Figure 1-3 — The sustained damage at day 7 after focal ischemia was represented by (A) 7-d Nissl staining and (B) the formation of glial scar indicated by anti-GFAP staining. Each slice was 50 μm. Download Figure 1-3, TIF file. [file enu-eN-NWR-0398-20-s03.tif]

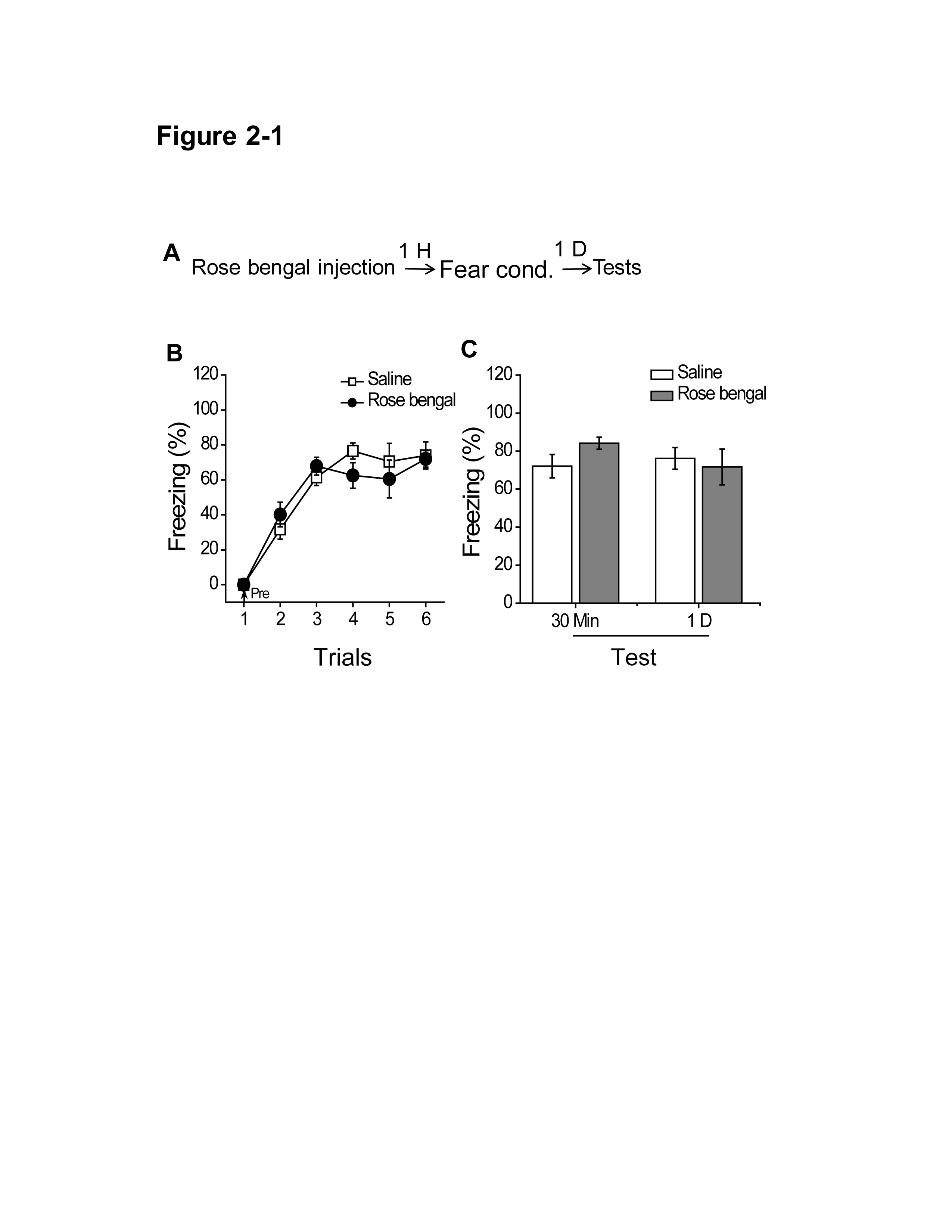

Supplement: Extended Data Figure 2-1 — The effects of Rose Bengal treatment on contextual fear formation. A, Schematic, rats were injected Rose Bengal (100 mg/kg, i.p.) 1 h before conditioning, and saline (10 ml/kg, i.p.) as control. B, Learning curve during conditioning (repeated measure ANOVA: F(1,8) = 0.08, p = 0.776). C, 30-min and 1-d dependent contextual tests (t test: 30 min, p = 0.156; 1 d, p = 0.725). Control, n = 5, Rose Bengal, n = 5. Mean ± SEM for each bar. Download Figure 2-1, TIF file. [file enu-eN-NWR-0398-20-s04.tif]

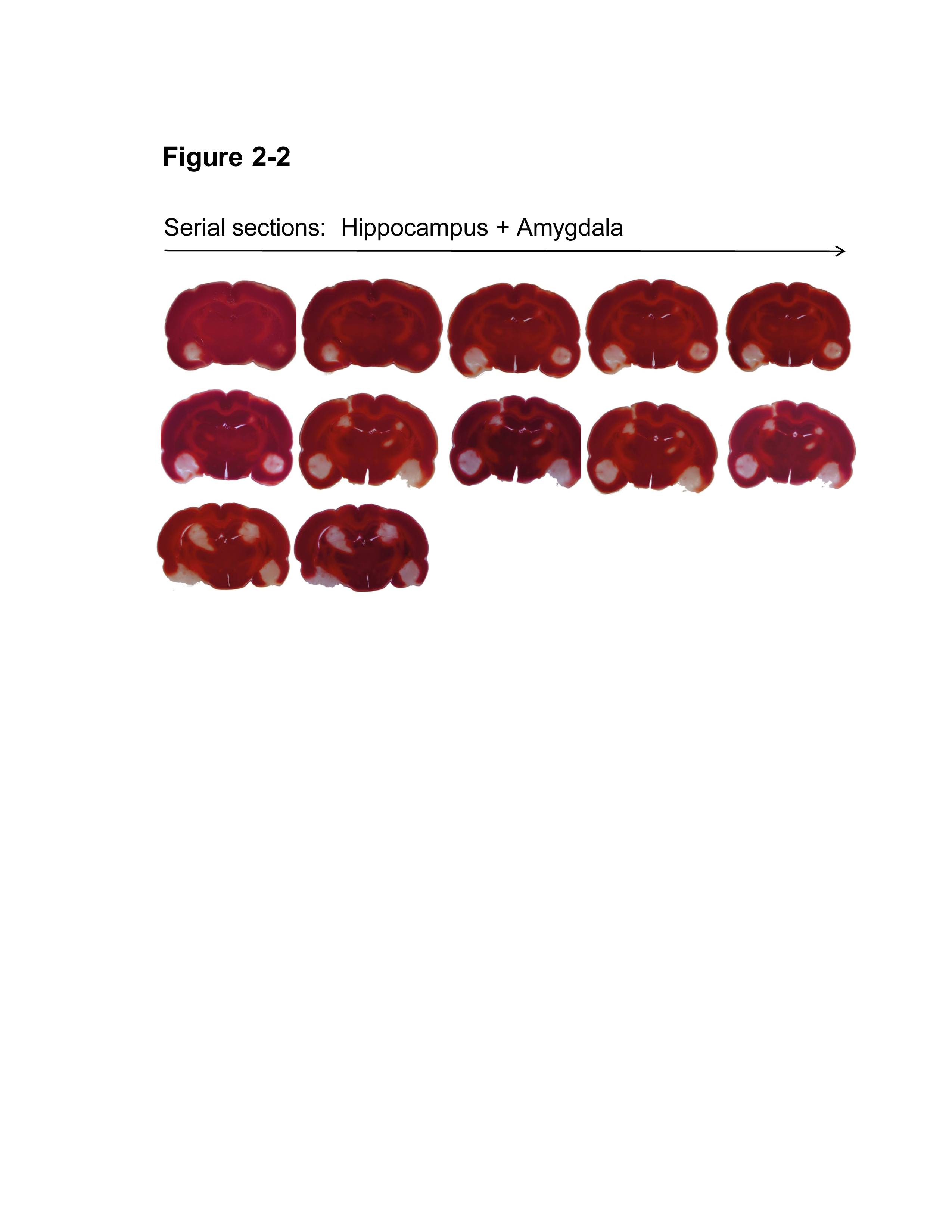

Supplement: Extended Data Figure 2-2 — Brain sections with TTC staining from a single rat one with 1-d ischemia in both hippocampus and amygdala. Each slice was 400 μm. Download Figure 2-2, TIF file. [file enu-eN-NWR-0398-20-s05.tif]

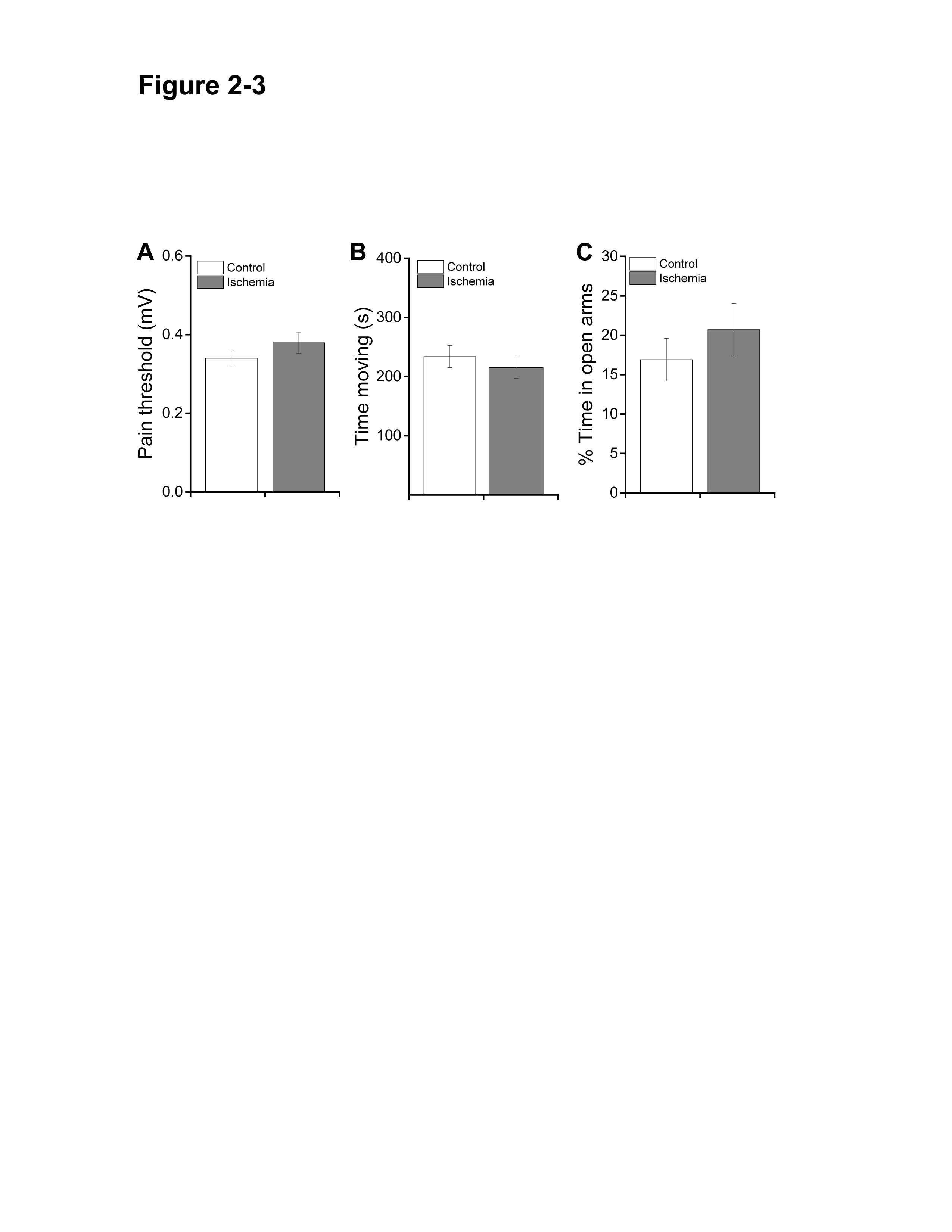

Supplement: Extended Data Figure 2-3 — The effects of 5-h ischemia in both the hippocampus and amygdala on other behaviors. A, Pain threshold test in fear conditioning box (control, n = 10 rats, ischemia, n = 9 rats; T = 2, p = 0.062, t test). B, Total time moving during EPM test (control, n = 10, ischemia, n = 9; T = 0.679, p = 0.502, t test). C, The percentage of time spend in open arms during EPM test (control, n = 10, ischemia, n = 9; T = 0.846, p = 0.409, t test). Mean ± SEM for each bar. Download Figure 2-3, TIF file. [file enu-eN-NWR-0398-20-s06.tif]

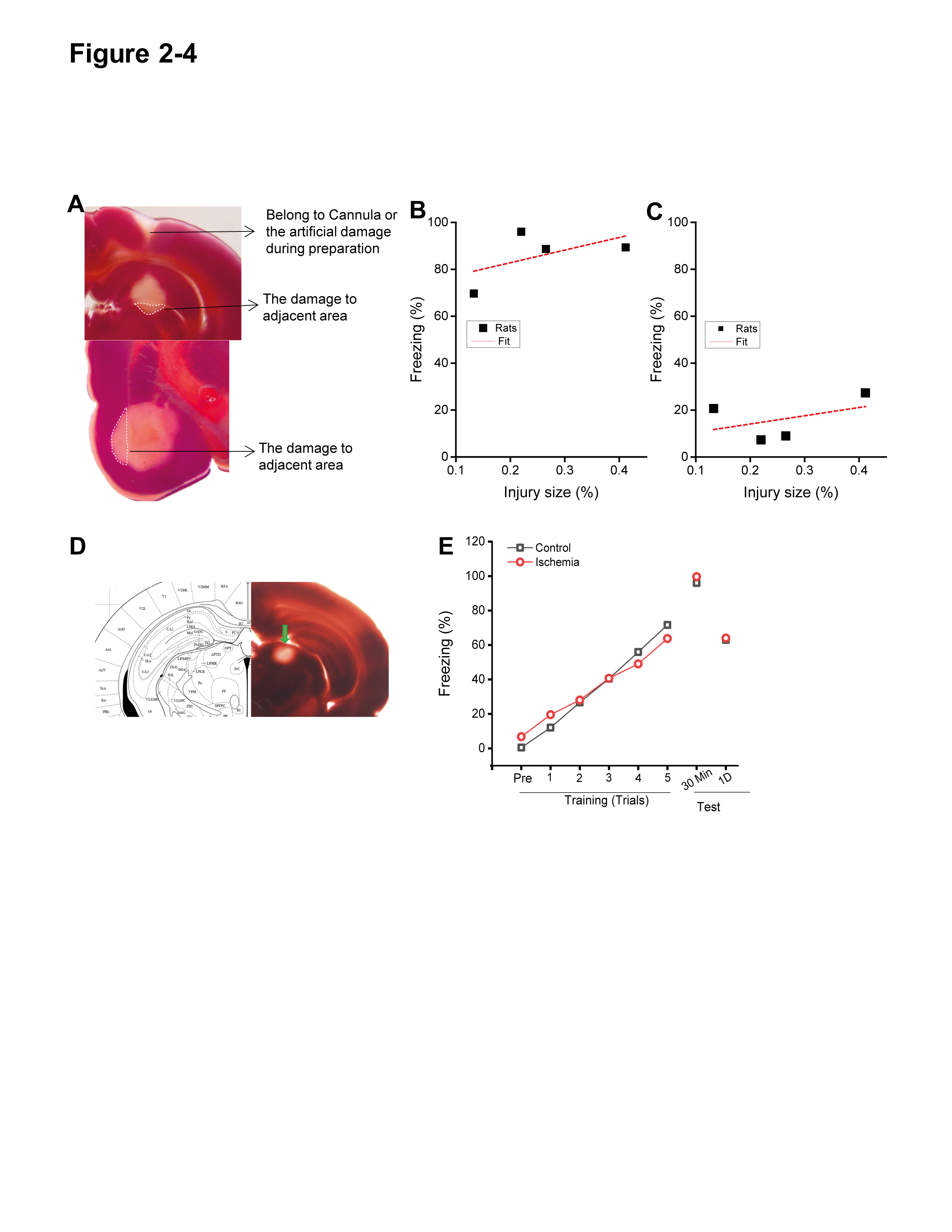

Supplement: Extended Data Figure 2-4 — The effects of the injury size at adjacent areas on the freezing behaviors. A, The white dash line indicated the damage to the adjacent areas out of the region of interest (ROI). Each slice was 400 μm. There was no correlation between the injury size of adjacent areas and the freezing time during the (B) 30-min (r = 0.003, p = 0.45) and (C) 1-d (r = 0.22, p = 0.57) memory test (n = 4). D, E, One rat with focal ischemia at the out of the ROI (the arrow) showed the same fear acquisition ability to its literature control. Download Figure 2-4, TIF file. [file enu-eN-NWR-0398-20-s07.tif]

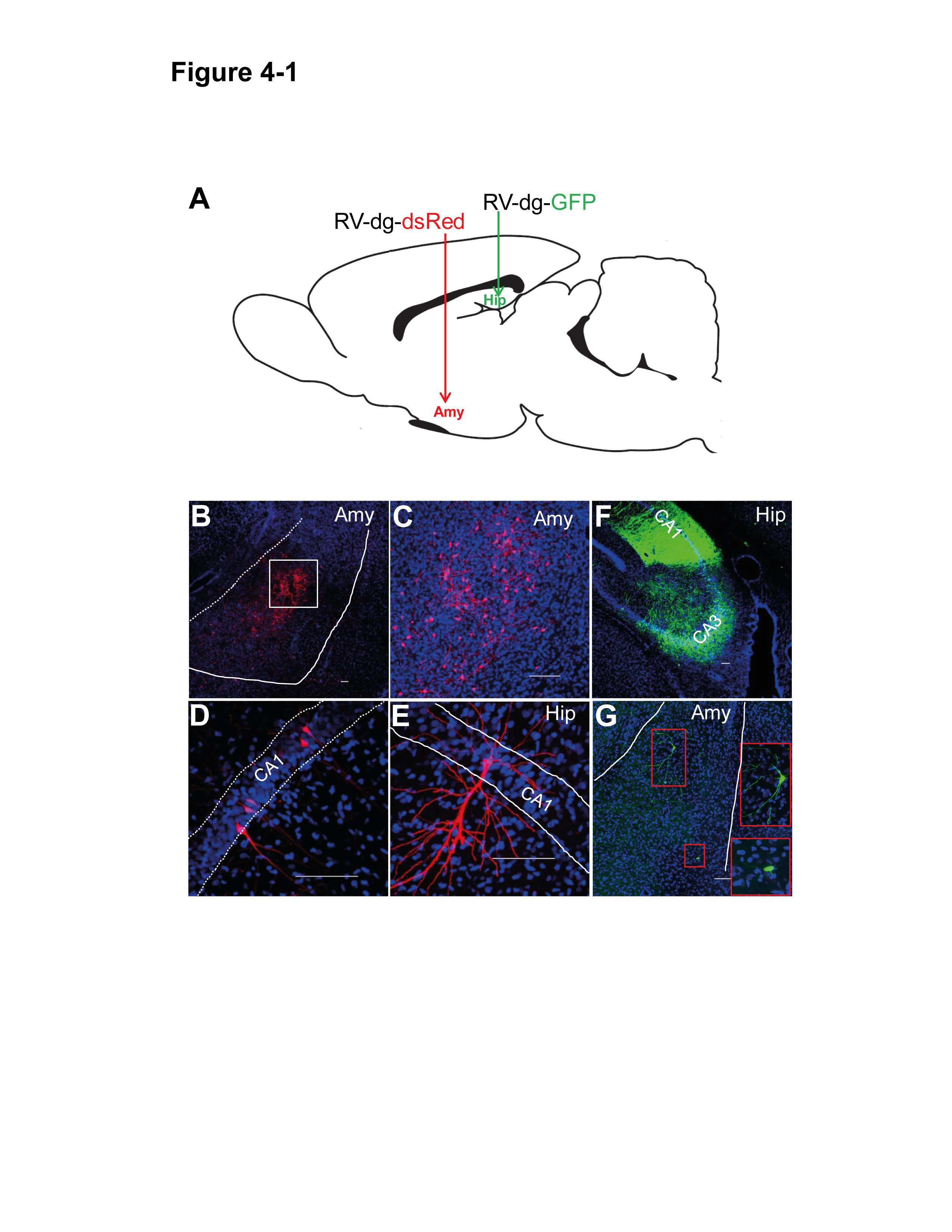

Supplement: Extended Data Figure 4-1 — The retrograde tracing of rabies virus in hippocampus or amygdala. A, Schematic: a non-transsynaptic rabies virus carried the GFP (RV-dg-GFP) as the reporter was injected into the unilateral hippocampus and a rabies virus carried the Dsred (RV-dg-dsRed) as the reporter was injected into the unilateral amygdala. B, C, The dsRed expression in the local injection site of the amygdala. D, E, The dsRed-positive cells expressed in the hippocampus. F, The GFP expression in the local injection site of the hippocampus. G, the GFP-positive cells expressed in the amygdala. Amy, amygdala; Hip, hippocampus. Each slice was 40 μm, confocal microscope scanning. All scale bars were 100 μm. Download Figure 4-1, TIF file. [file enu-eN-NWR-0398-20-s09.tif]
